# Supplementary material for: Modelling the effects of diurnal temperature variation on malaria infection dynamics in mosquitoes
Source: Commun Biol. 2025 Apr 8;8:581. doi: 10.1038/s42003-025-07949-5 (PMC11979013; doi:10.1038/s42003-025-07949-5)
Supplement: Supplementary file 1 — Supplementary information [file 42003_2025_7949_MOESM1_ESM.pdf]

Supplementary information

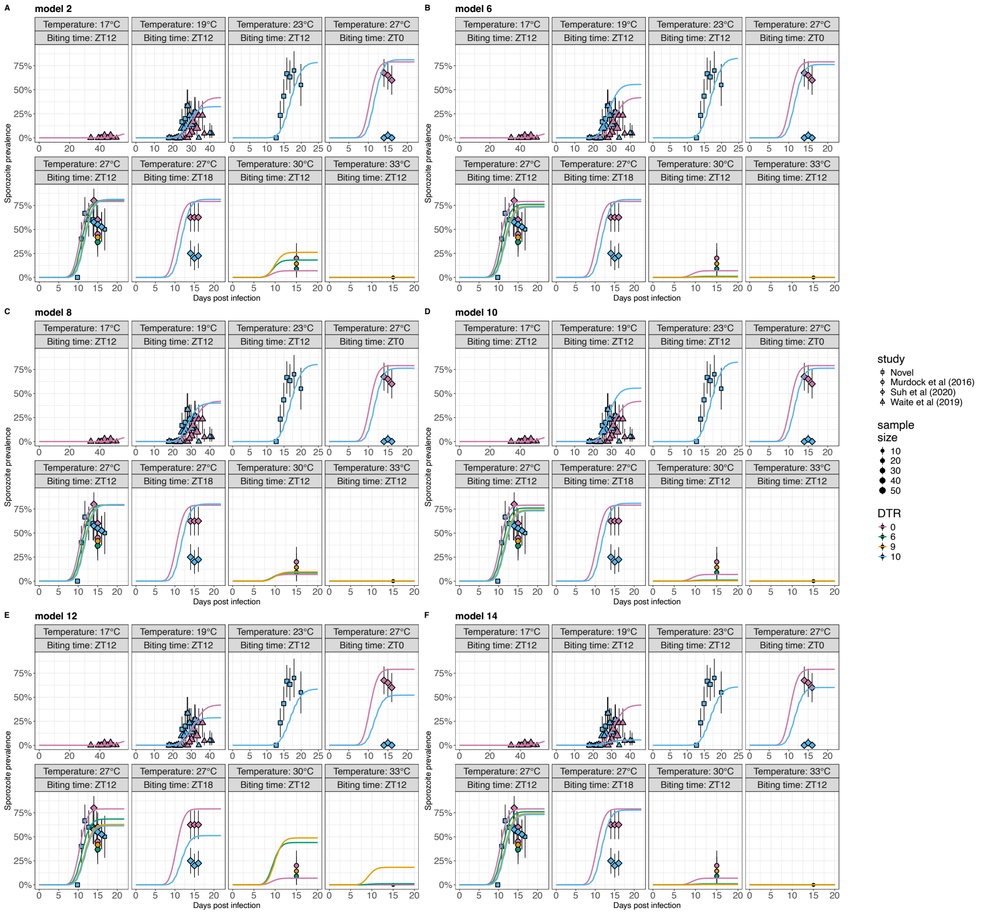

Fig S1. Cumulative sporozoite prevalence membrane feeding assay simulations for the different methods to estimate the HMTP. Sporozoite prevalence was simulated by the best fitting model, in which the PDR (β) was determined instantaneously by temporal changes in temperature. The method to determine the HMTP (c) is shown in the panel titles. The points show the observed sporozoite prevalence values and the vertical lines show the 95% binomial confidence intervals. The top facet shows the temperature, the lower facet shows the biting time, the shape of the points indicate the study the data was obtained from, and the colour shows the DTR. For all plots biting time is in Zeitgeiber time (CT), where 12:00 is the beginning of night time.

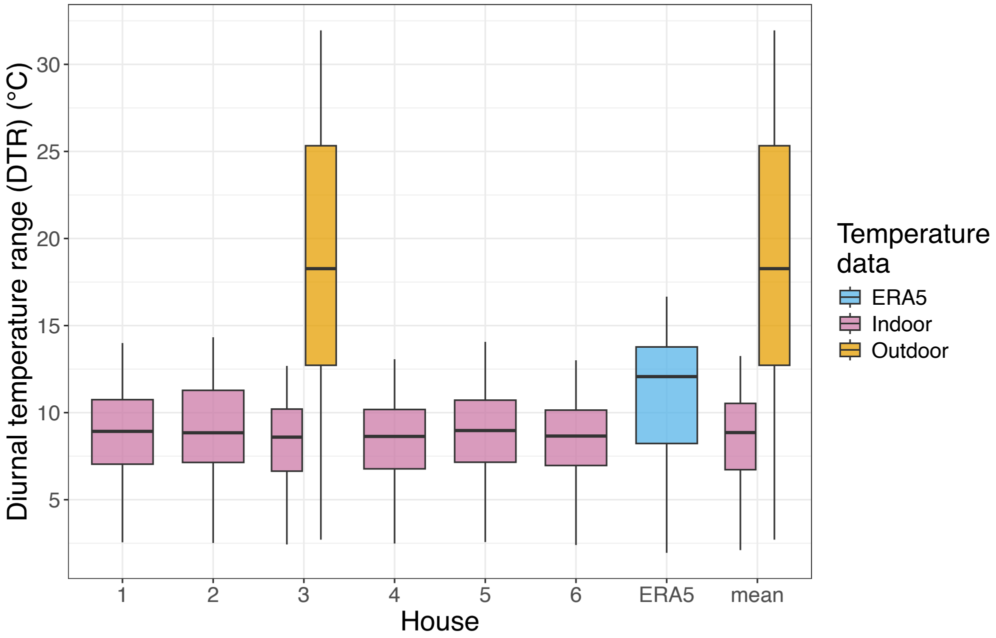

Fig S2. Differences in the daily diurnal temperature ranges (DTRs) between different sources of temperature data. The Tukey-style box plots show the median (horizontal line), 25th percentile (Q1; lower hinge), 75th percentile (Q3; upper hinge), Q1 minus 1.5 times the interquartile range (lower whisker) and Q3 plus 1.5 times this interquartile range (upper whisker), for the daily DTRs between 2020. Note that for the microclimatic indoor and outdoor temperatures data is only available for a subset of the year.

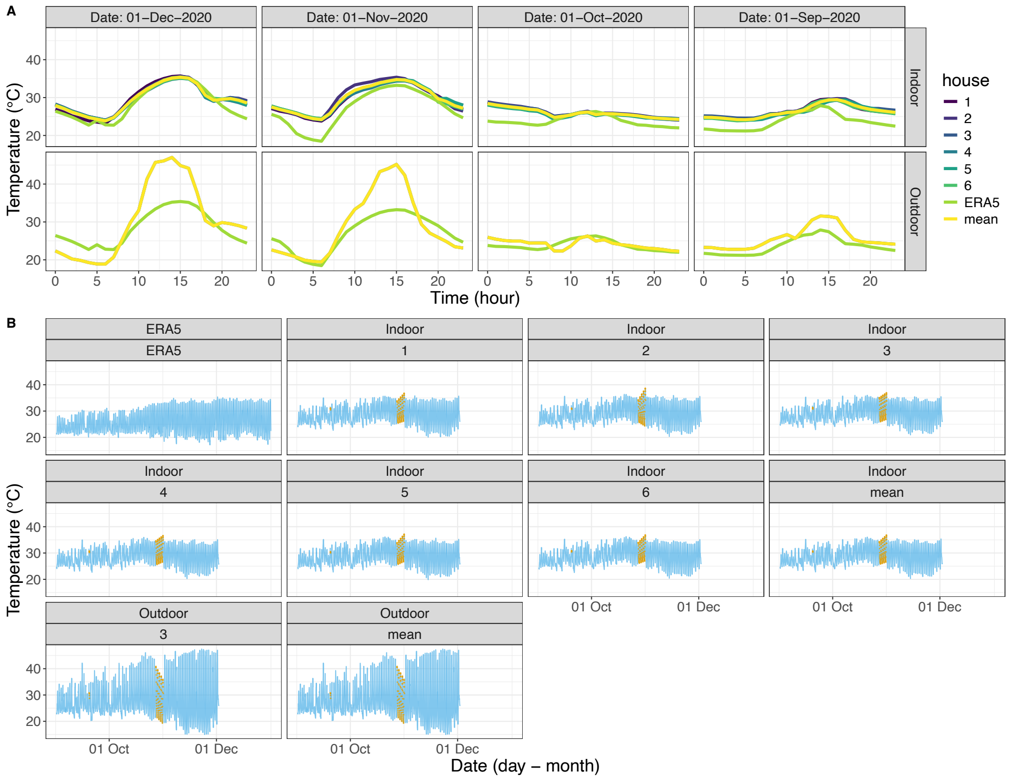

Fig S3. Microclimatic temperature logger data from Tiefora, Burkina Faso. (A) The hourly changes in temperature for 6 different experimental huts for the first day of each month are shown. The column facets indicate the date and the column facets indicate whether the temperature logger was placed indoors or outdoors of experimental trial huts. Outdoor temperature data was only available for a single house (3). The mean indoor temperature for all the houses is shown by the yellow line. The ERA5 19 re-analysis 2m air temperature data[1] for the grid square containing Tiefora was also plot; these estimates were assumed to be the same for both the indoors and outdoors. (B) Long-term trends in the hourly temperature data. Orange points indicate the hours for which the hourly temperature data was interpolated.

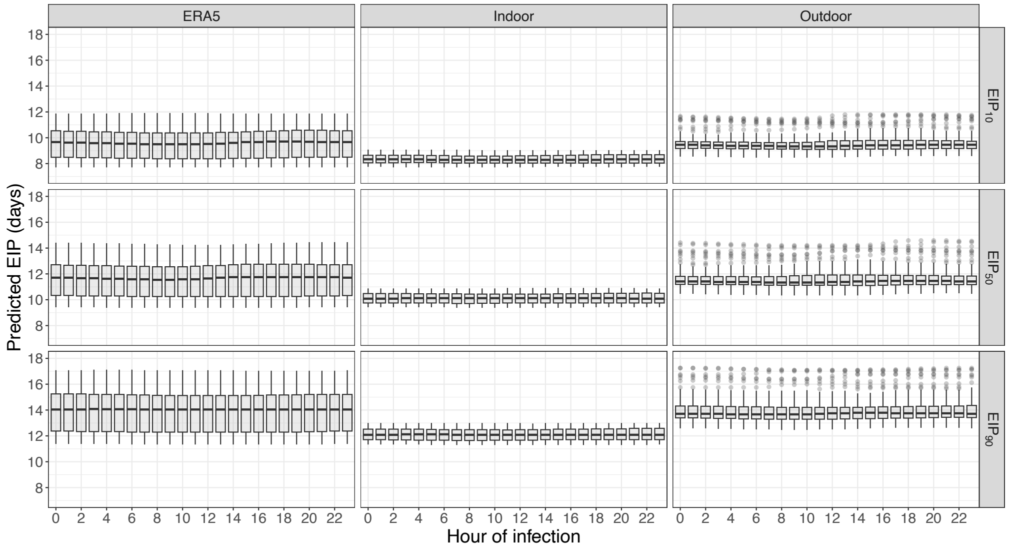

Fig S4. The effects of the time of infection on the model-derived EIP. The Tukey-style box plots show the median (horizontal line), 25th percentile (Q1; lower hinge), 75th percentile (Q3; upper hinge), Q1 minus 1.5 times the interquartile range (lower whisker) and Q3 plus 1.5 times this interquartile range (upper whisker), for all predicted values in the year 2020. Note that for the microclimatic indoor and outdoor temperatures predicted values are only available for a subset of the year. The hour of infection is not in Zeitgeiber time.

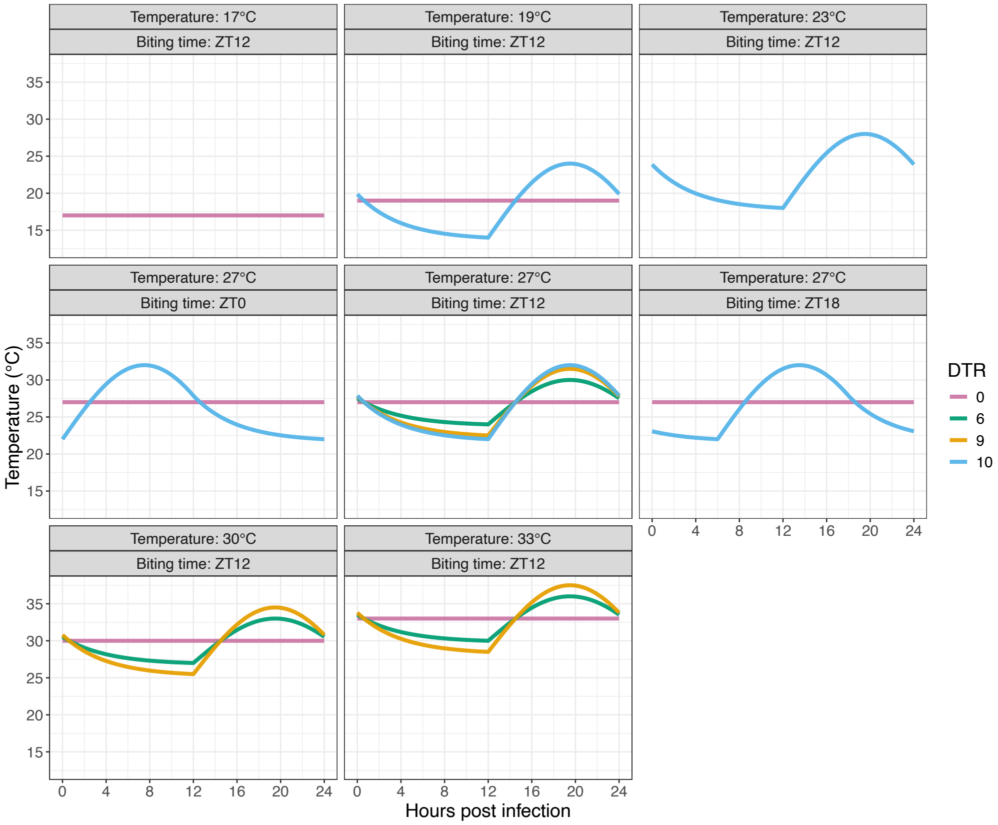

Fig S5. Temperature profiles according to the Parton-Logan model given differences in the diurnal temperature range (DTR) and time of infection (biting time). ZT refers to Zeitgeiber time where 12 is the beginning of night time and 0 the beginning of day time.

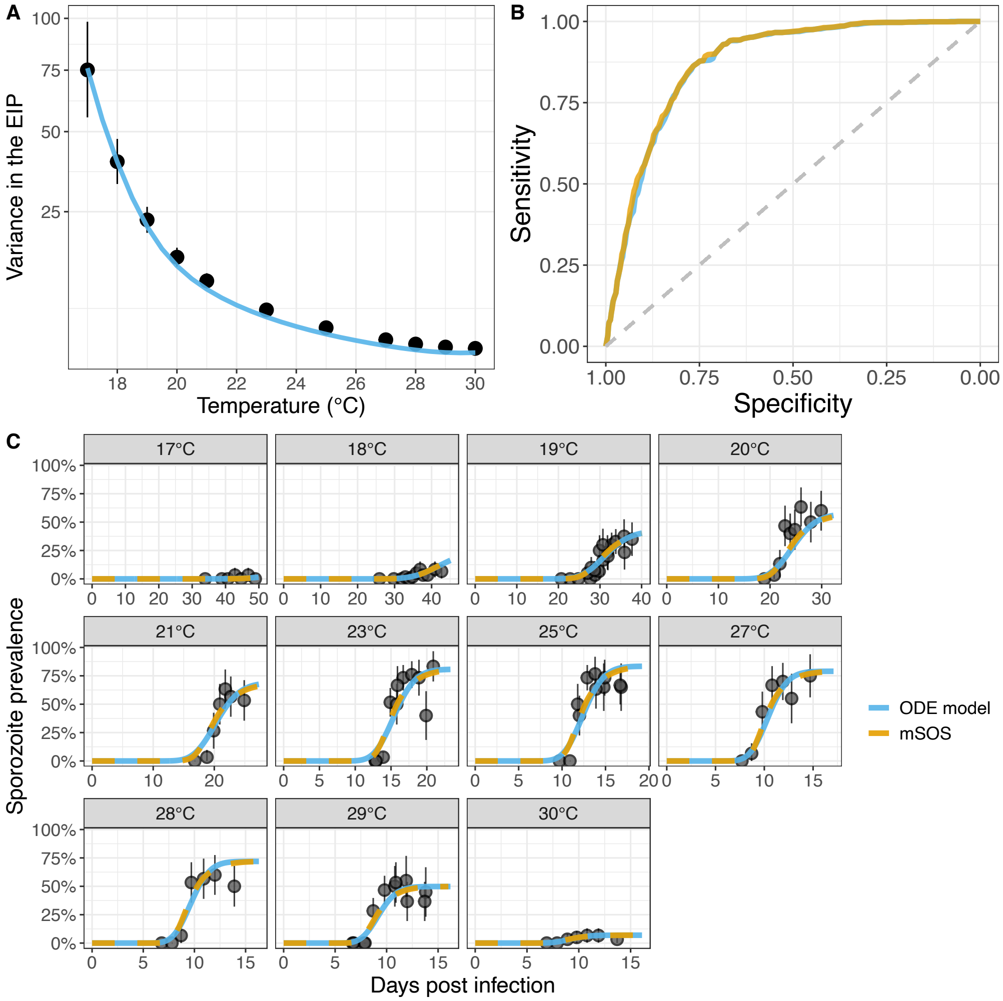

Fig S6. Estimating the extrinsic incubation period using the linear chain trick (LCT) (A) The relationship between the Erlang distribution variance with a shape parameter of 47 (blue line) and the EIP distribution estimated by mSOS[2] (points). Vertical lines show the 95% credible intervals. (B) ROC curves of the different methods to predict the presence of sporozoites in mosquitoes dissected during SMFAs in Suh et al[2]. Both model lines overrun each other indicating a comparable fit to data (C) The predicted sporozoite prevalence during SMFAs for the constant temperature data. Points show the sampled sporozoite prevalence values from Suh et al[2]. Vertical lines show the 95% binomial confidence intervals. Again both the models overrun each other.

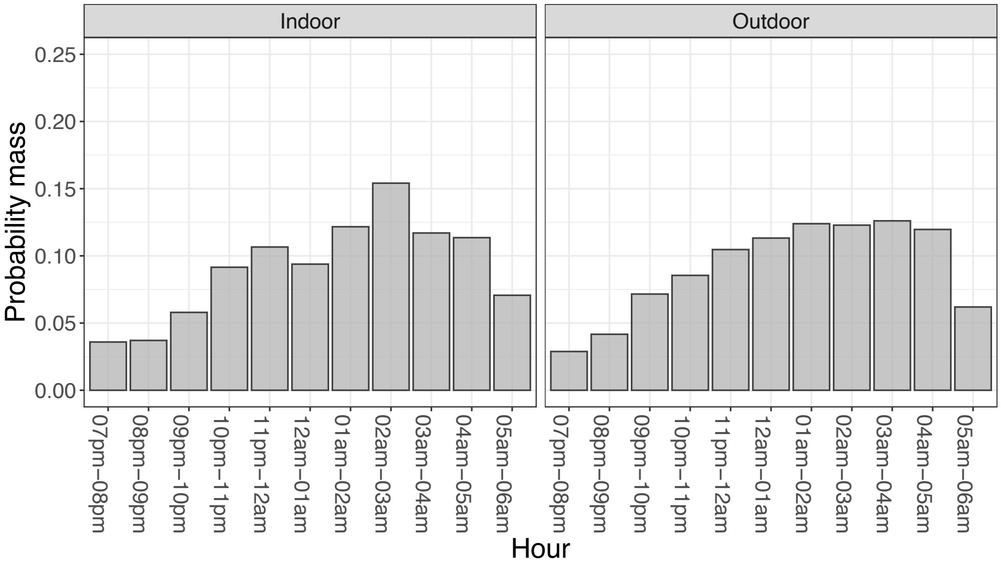

Fig S7. Probability distribution of different mosquito biting times. Data for *A. gambiae sl* from nearby villages to the temperature data and entomological data (presented in Figure 3 of the main text) were obtained from[3] and aggregated by village and date. Biting times of n=1799 mosquitoes collected from two villages between October 2016 and December 2019 [3] were included.

Table S1. Adapted Ross-Macdonald model fits to the sampled sporozoite prevalence in the Cascades region of Burkina Faso. The sensitivity of the model estimates to different human Recovery rate (r; per person per day) and mosquito biting rates (a; per mosquito per day) was also assessed. The area under the receiver operating characteristic curve (AUC) for sporozoite presence (1: sporozoite positive, 0: sporozoite negative) and mean absolute error (MAE) for the absolute sporozoite prevalence percent estimates was assessed in the data for 2018 and the model was fitted to data prior to 2018.

**Model**	r	a	**HMTP scaling parameter (ψ)**	**Log-likelihood**	**Sporozoite prevalence AUC**	**Sporozoite prevalence MAE**
DTR-dependent	1/50	1/2	0.03	-7.42	0.6	2.94%
DTR-independent	1/50	1/2	0.01	-7.18	0.58	3.2%
Constant	1/50	1/2	0.04	-7.17	0.62	3.18%
DTR-dependent	1/100	1/2	0.03	-7.41	0.62	2.91%
DTR-independent	1/100	1/2	0.01	-7.16	0.57	3.14%
Constant	1/100	1/2	0.04	-7.15	0.62	3.21%
DTR-dependent	1/50	1/3	0.05	-7.42	0.6	2.94%
DTR-independent	1/50	1/3	0.02	-7.18	0.58	3.2%
Constant	1/50	1/3	0.05	-7.17	0.62	3.18%
DTR-dependent	1/100	1/3	0.05	-7.41	0.62	2.91%
DTR-independent	1/100	1/3	0.02	-7.16	0.57	3.14%
Constant	1/100	1/3	0.05	-7.15	0.62	3.21%

Table S2. Experimental replicates and dissection times for the standard membrane feeding assay (SMFA) testing data. DPI refers to the days post infection. All mosquitoes were *A. gambiae*, which were fed blood infected with *P. falciparum*.

Temperature (°C)	Diurnal temperature range (DTR) (°C)	Biting time	Gametocyte density	DPI: sporozoite dissection	Sample size	Reference
19	10	ZT12	0.126%	17.8, 20.9, 21.8, 22.7, 23.7, 24.9, 25.8, 26.7, 27.7, 28.8, 29.8 & 31.8	360	Novel
23	10	ZT12	0.126%	12.7, 13.9, 14.9, 15.8, 16.7, 17.9 & 19.9	190	Novel
27	10	ZT12	0.126%	9.8, 10.8, 11.7, 12.7, 13.8, 14.7 & 16.8	190	Novel
27	6	ZT12	8%	15	38	Murdock et al (2016)
27	9	ZT12	8%	15	36	Murdock et al (2016)
30	0	ZT12	8%	15	25	Murdock et al (2016)
30	6	ZT12	8%	15	22	Murdock et al (2016)
30	9	ZT12	8%	15	21	Murdock et al (2016)
33	0	ZT12	8%	15	8	Murdock et al (2016)
33	6	ZT12	8%	15	8	Murdock et al (2016)
33	9	ZT12	8%	15	1	Murdock et al (2016)
27	0	ZT0	8%	14, 15 & 16	120	Suh et al (2020)
27	0	ZT12	8%	14, 15 & 16	120	Suh et al (2020)
27	0	ZT18	8%	14, 15 & 16	120	Suh et al (2020)
27	10	ZT0	8%	14, 15 & 16	120	Suh et al (2020)
27	10	ZT12	8%	14, 15 & 16	120	Suh et al (2020)
27	10	ZT18	8%	14, 15 & 16	120	Suh et al (2020)
17	0	ZT12	2-4%	34, 39, 40, 41, 43, 45, 47, 49 & 51	268	Waite et al (2020)
19	0	ZT12	2-4%	22, 23, 27, 28, 29, 30, 31, 32, 33, 34, 36, 37, & 41	396	Waite et al (2020)
19	10	ZT12	2-4%	18, 19, 21, 22, 23, 24, 25, 26, 27, 28, 29, 30, 32, 34, & 40	489	Waite et al (2020)

References

1. Hersbach, H. *et al.* The ERA5 global reanalysis. *Quarterly Journal of the Royal Meteorological Society* **146,** 1999–2049 (2020).
2. Suh, E. *et al.* Estimating the effects of temperature on transmission of the human malaria parasite, *Plasmodium falciparum. Nature Communications* **15,** 3230 (2024).
3. Guglielmo, F. *et al.* Quantifying individual variability in exposure risk to mosquito bites in the Cascades region, Burkina Faso. *Malaria Journal* **20,** 44 (2021).
